# BEYOND REDLINING: LAW, PROPERTY VALUES, RACIAL INEQUITIES, AND LTSS

**DOI:** 10.1093/geroni/igad104.0159

**Published:** 2023-12-21

**Authors:** Lindsey Smith

**Affiliations:** Oregon Health & Science University, Portland, Oregon, United States

## Abstract

This presentation will provide background regarding the social construction of property values and the interactive role that public and private institutions have played in creating racial disparities in property values. I will discuss known and proposed mechanisms through which this form of structural racism affects access to long-term services and supports (LTSS) and implications for the health of older adults and their care partners, presenting a specific example from my own research, which found that assisted living communities are located in 30% of communities in America. However, among communities with the highest percent Black population in their county, 31% of those located in counties with a low gap in Black-White economic mobility had access to assisted living, while less than 15% of those located within a county with a high Black-White gap in economic mobility had such access. I will describe how policymakers can use law to address racial disparities in geographic access by developing policies that 1) provide financial incentives for private investors to develop assisted living and other forms of LTSS in neighborhoods that otherwise wouldn’t see development due to low property values; 2) prioritize the location of new buildings in historically marginalized communities; 3) implement anti-displacement measures; 4) incorporate community input and engagement; and 5) ensure accessibility and affordability. Finally, I will describe how policymakers may consider the various actors and institutions that shape the housing market and how they interact with each other to produce outcomes that may reinforce or challenge structural racism.

